# Laparoscopic-assisted hydrostatic reduction of pediatric intussusception: a prospective study from a single tertiary center

**DOI:** 10.1186/s12893-025-03288-8

**Published:** 2025-11-14

**Authors:** Ahmed Abdelmhaimen Elhaddad, Youssef Youssef Aboshosha, Abdelmotaleb Effat Ebeid, Mohamed Ali Shehata

**Affiliations:** Pediatric Surgery Department, Faculty of Medicine, Tanta University, El-Geish Street, Tanta, 31257 Egypt

**Keywords:** Intussusception, Laparoscopy, Hydrostatic reduction, Pediatric surgery, Minimally invasive techniques

## Abstract

**Background:**

After failed ultrasound-guided hydrostatic reduction (USGHR), operative options vary. We evaluated a protocolized laparoscopic-assisted hydrostatic reduction (LAHR) pathway as an escalation strategy.

**Methods:**

This prospective study included 242 children with intussusception treated at a single tertiary center between January 2023 and December 2024. All children received standardized USGHR (≤ 3 attempts in one session). Failures proceeded to LAHR under standardized hydrostatic pressure (80–100 cm H₂O); persistent non-reduction or ischemia/lead point prompted mini-laparotomy and resection. Primary outcome was LAHR success; secondary outcomes included early recurrence (≤ 48 h), complications, time to feeds, and length of stay (LOS). Discharge was milestone-based.

**Results:**

Of 242 children, USGHR succeeded in 189 (78.1%); 53 (21.9%) underwent LAHR. LAHR succeeded in 45/53 (84.9%); 8/53 (15.1%) required laparotomy. No intraoperative perforations occurred. Complications after LAHR were low: intraperitoneal infection 1.9%, ileus 3.8%, wound infection 1.9%. Time to enteral feeding was 8.2 ± 1.7 h after uncomplicated LAHR and 47.5 ± 5.8 h with resection. Mean LOS was 1.54 ± 0.36 days after uncomplicated LAHR, 4.25 ± 0.71 days with resection, and 1.95 ± 1.07 days overall. No early (≤ 48 h) recurrences were detected on routine in-hospital surveillance.

**Conclusions:**

A pressure-standardized LAHR pathway is a safe, effective escalation after failed USGHR, achieving high reduction success with low morbidity, rapid feeding, short LOS, and no early recurrences. These reproducible methods offer actionable benchmarks for multicenter comparison; confirmation in larger, comparative cohorts with longer follow-up is warranted.

**Trial registration:**

The Pan African Clinical Trial Registry (pactr.samrc.ac.za) database, PACTR202508726005103, Registration date: 14 August 2025.

## Introduction

 Intussusception is a leading cause of bowel obstruction in children. Diagnosis is primarily established with abdominal ultrasonography, which has high sensitivity and specificity and provides real-time assessment of the intussusceptum and associated complications. It is widely accepted as the first-line, gold-standard imaging modality. Contrast enema may also be used, serving both diagnostic and therapeutic roles in many centers. Computed tomography is rarely indicated and is generally reserved for atypical or complicated cases, particularly in older children or when ultrasound findings are equivocal [1–3]. Approximately 85% of these cases are reduced via pneumatic or hydrostatic enema [4].

Radiological guided reduction of intussusception (air or saline enema) is considered the first-line treatment, with an average success rate 80% (range 40%-90%) [1, 2]. Surgical intervention is required when enema reduction fails or when hemodynamic instability and/or peritonitis is present, typically in 10 to 20% of cases [3]. Traditionally, unsuccessful radiological reduction has been followed by laparotomy [5].

The introduction of laparoscopy in pediatric surgery has added another dimension to management. Its use has increased significantly over the past two decades [6]. Initially utilized as a diagnostic modality after failed hydrostatic reduction, laparoscopy is now also used as definitive treatment [7, 8].

Reported advantages of the laparoscopic over the open approach include less postoperative pain, fewer wound complications, minimal scarring, shorter hospital stay, and earlier return to normal activities [9, 10].

The purpose of this study was to evaluate the benefit of laparoscopy in guiding hydrostatic reduction of intussusception and evaluation of the reduced mass with regard to perforation, ischemic signs or other pathology.

## Materials and methods

### Study design and setting

This prospective study included 242 pediatric patients with clinically and ultrasonographically confirmed intussusception treated at the Pediatric Surgery Unit, General Surgery Department, Tanta University Hospitals, Tanta, Egypt; from January 2023 to December 2024.

All patients underwent ultrasound-guided hydrostatic reduction (USGHR): 189 were successfully reduced, and 53 proceeded to laparoscopic-assisted hydrostatic reduction (LAHR) under general anesthesia after failed USGHR (45 had successful LAHR; 8 required mini-laparotomy). Written informed consent was obtained from parents/guardians. The study was approved by the University Ethics Committee and registered as a clinical trial.

A formal sample size calculation was not performed. All eligible patients treated during the study period were included to reflect real-world practice at a high-volume tertiary center, consistent with feasibility/pilot methodology.

### Eligibility criteria

Inclusion criteria were all pediatric intussusception cases. Exclusion criteria were unstable general condition, contraindication of laparoscopic surgery (e.g. cardiac disease), patients with marked abdominal distension, and those with signs of peritonitis.

### Management protocol

Ultrasound-guided hydrostatic reduction (USGHR) was the first-line approach, supervised by senior pediatric radiologists and performed using a standardized institutional protocol. Each attempt lasted approximately 3–5 min, followed by a 5-minute interval; a maximum of three attempts was allowed. After three failed attempts, patients were prepared and transferred for LAHR, typically initiated within 30–45 min [1, 11]. This was an institutional protocol practiced consistently across the study period, and not subject to individual surgeon preference. No cases were directed to open surgery directly after failed USGHR without attempting LAHR.

### Data collection

**Fig. 1:**
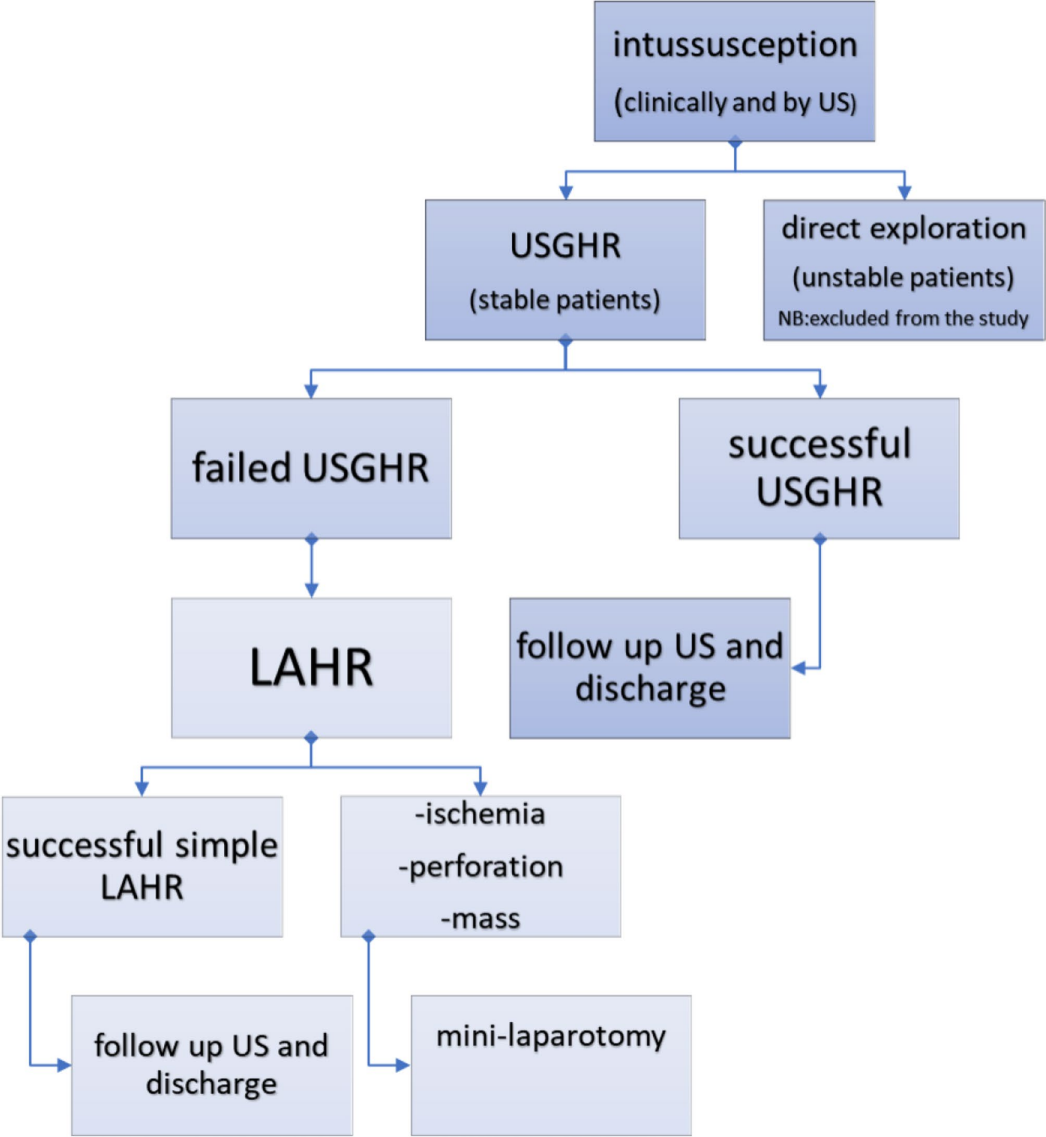
Algorithm summarizes the management pathway

All patients were subjected to the following: demographic data collection from each patient including (age, sex, duration of symptoms), key clinical presentation including (vomiting, red currant jelly stool, intestinal obstruction, shock), and anatomical site of intussusception were collected for all patients and analyzed. The site of the intussusceptum was documented anatomically in relation to the colon (ileocecal/ascending, transverse, descending, sigmoid, or rectosigmoid) rather than by abdominal quadrant location. Parameters related to hydrostatic reduction—number of attempts, duration and intervals between attempts, and subsequent laparoscopic management—were prospectively documented and analyzed. Routine clinical and laboratory assessments were performed for perioperative care but were not included in the data analysis. Figure 1, Algorithm summarizes the management pathway.

### Laparoscopic-assisted hydrostatic reduction (LAHR) technique

**Fig. 2:**
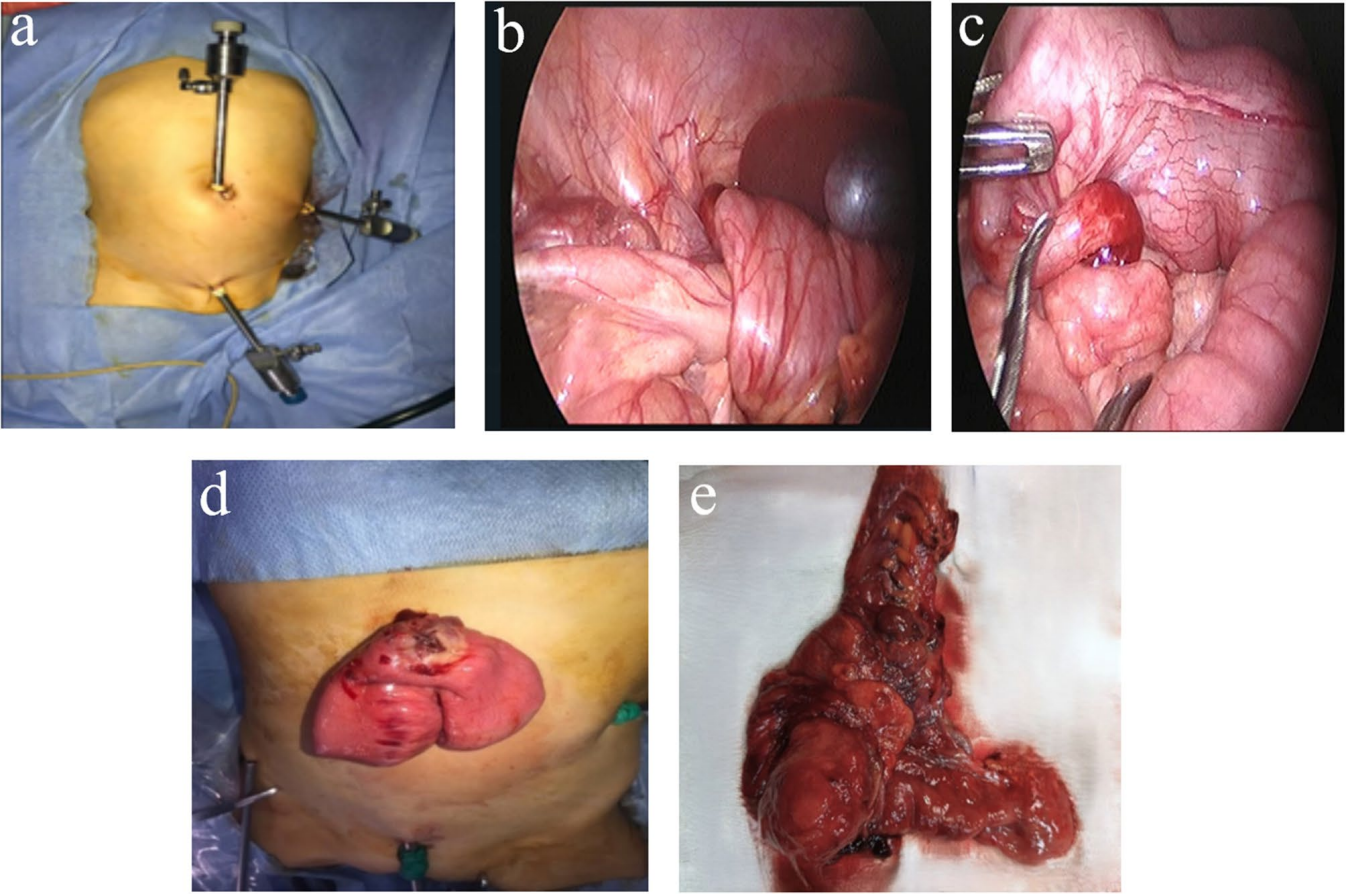
Intraoperative steps and key findings during laparoscopic-assisted hydrostatic reduction (LAHR) for intussusception. **a** Trocar placement, **b** Subhepatic position of the intussusception mass identified on laparoscopic inspection, **c** Inflamed appendix acting as a pathological lead point, **d** Exteriorization of the involved bowel through a limited umbilical incision following failed laparoscopic reduction, **e** Gangrenous ileocecal segment requiring resection and anastomosis

Following induction of general anesthesia, patients were positioned supine, and hydrostatic reduction was prepared before laparoscopic setup. A 24 F Foley catheter was secured in the rectum, and a saline bag was set to yield 80–100 cm H₂O hydrostatic pressure (bag height above the anal verge), adjusted within this range to maintain luminal filling under direct vision without over-distension. Pneumoperitoneum was established through an umbilical 5 mm port (8–10 mmHg), with two additional working trocars as needed according to the site of the intussusception. Under laparoscopic vision, hydrostatic reduction was initiated, when necessary, gently assisted with atraumatic graspers. After reduction, the involved bowel segment was inspected carefully for viability and to exclude perforation or pathological lead points. Conversion to mini-laparotomy was performed in cases of failed reduction or nonviable bowel for resection and anastomosis (Fig. 2).

### Outcome definitions

The primary outcome: LAHR success, defined as complete reduction of the intussusception mass under laparoscopic visualization and restoration of normal bowel appearance and continuity, without conversion to mini-laparotomy or bowel resection. A successful case also included the absence of intraoperative findings requiring resection (e.g., perforation, necrosis, or tumor).

Secondary outcomes: early recurrence of intussusception and postoperative complications.

- Early recurrence: recurrence within 48 h of reduction, confirmed by ultrasound.
- Postoperative infection: purulent fluid in drain or intraperitoneal space with systemic signs such as fever > 38 °C or leukocytosis), or ultrasound evidence; treated with appropriate antibiotics and supportive measures.
- Postoperative ileus: abdominal distension, delayed return of bowel function (no flatus or stool > 48 h), and radiologic evidence of air-fluid levels.
- Wound infection: localized erythema, warmth, and/or purulent discharge at the trocar or mini-laparotomy sites.

### Postoperative care

All patients were recovered and admitted to the ward; they remained NPO and received IV 3rd -generation cephalosporin (100 mg/kg/dose), metronidazole (7.5 mg/kg/dose), and IV paracetamol (7.5 mg/kg/dose) as postoperative analgesia. At our institution, all patients routinely received short-course antibiotic prophylaxis (of intravenous third-generation cephalosporin and metronidazole). This approach was based on institutional policy to reduce potential risks of bacterial translocation, although we recognize that in many centers, antibiotics are reserved for complicated cases [10, 12].

Oral intake followed a milestone-based protocol and typically began within 6–12 h post-procedure, advancing as tolerated. Follow-up by U/S before discharge was conducted to detect recurrence or complications. Discharge was milestone-based: patients were discharged once afebrile, hemodynamically stable, with adequate analgesia and tolerance of enteral feeds.

Discharge medications were oral antibiotics according to weight, oral paracetamol on demand. Routine oral antibiotics on discharge were prescribed according to institutional policy. Although uncomplicated intussusception typically does not require post-reduction antibiotic therapy in many international protocols, our local practice involves administering a short oral course at discharge. This policy is primarily driven by concerns regarding potential bacterial translocation, and by institutional infection-control strategies in a setting with variable outpatient follow-up reliability. We acknowledge that this differs from standard selective approaches recommended elsewhere.

### Statistical analysis

Analyses were performed using SPSS v28 (IBM, Armonk, NY, USA). Normality was assessed with the Shapiro–Wilk test and histograms. Parametric data are presented as mean ± standard deviation (SD); non-parametric data as median and interquartile range (IQR); categorical variables as frequency and percentage, with 95% confidence intervals for proportions calculated using the exact (Clopper–Pearson) method. Given the single-arm, descriptive design evaluating outcomes after LAHR following failed USGHR, no hypothesis testing (p-values) was performed; no control group was available for comparative statistics.

## Results

### Demographic and clinical characteristics

The study included 242 patients. The median age was 10 months (IQR 8–13.8), and 58.7% were male. Most patients presented with classic symptoms, including red currant jelly stool and signs of intestinal obstruction, while 18.2% were in hypovolemic shock on admission. The ileocecal/ascending colon was the most frequent anatomical site of intussusception, followed by the transverse and descending colon. Detailed baseline characteristics are summarized in Table 1.

**Table 1:** Demographics, preoperative clinical data and site of intussusception mass of the studied patients

Category	Count	Total	Percentage (%)	95% confidence interval
Age (months) Median (IQR)	14.1 ± 11.46 10 (8–13.75)	242		
Sex				
Male	142	242	58.68%	52.48% – 64.88%
Female	100	242	41.32%	35.12% – 47.52%
Preoperative clinical data				
Early with mild symptoms	133	242	54.96%	48.76% – 61.16%
Red currant jelly stool	145	242	59.92%	53.72% – 66.12%
Intestinal obstruction	153	242	63.22%	57.02% – 69.42%
Shock	44	242	18.18%	13.88% – 24.50%
Site of intussusception mass				
Ileocecal/ascending	149	242	61.57%	55.12% – 67.54%
Transverse colon	48	242	19.83%	15.30% – 25.02%
Descending colon	32	242	13.22%	9.38% – 18.09%
Sigmoid colon	9	242	3.72%	1.71% – 6.98%
Rectosigmoid	4	242	1.66%	0.91% – 4.17%

Data presented as mean ± SD, median or frequency (%)

### Success rates of USGHR and LAHR

Ultrasound-guided hydrostatic reduction was successful in the majority of patients 78.1% (189/242). Laparoscopic-assisted hydrostatic reduction (LAHR) was performed in 53 patients after failed enema reduction, with a success rate of 84.9% (45/53). Eight patients (15.1%) required resection and anastomosis. Postoperative complications were infrequent. Aggregate procedural outcomes are shown in Table 2.

**Table 2:** US-guided hydrostatic reduction (USGHR), failed reduction and LAHR of the studied patients, and postoperative complications

Category	Count	Total	Percentage (%)	95% confidence interval
Successful USGHR	189	242	78.09%	72.73% – 83.06%
-USGHR -One trial	126	189	66.67%	59.79% – 73.54%
-USGHR -Two trials	47	189	27.87%	19.05% – 31.22%
-USGHR -Three trials	16	189	5.46%	4.76% – 12.70%
LAHR	53	242	21.91%	16.94% – 27.27%
-Successful Simple LAHR	45	53	84.91%	72.95% – 92.15%
-Resection anastomosis	8	53	15.09%	7.85% – 27.05%
Postoperative Intraperitoneal Infection	1	53	1.89%	0.33% – 9.94%
Postoperative Ileus	2	53	3.77%	1.04% – 12.75%
Wound Infection	1	53	1.89%	0.33% – 9.94%

Data presented as frequency (%)

*LAHR* laparoscopic assisted hydrostatic reduction

### Postoperative outcomes and complications

No iatrogenic perforations occurred during laparoscopic manipulation of the saline-filled colon, supporting the safety of this technique when performed under direct laparoscopic guidance [10, 12].

Under the milestone-based discharge pathway, the mean length of hospital stay (LOS) after LAHR was 1.95 ± 1.07 days (*n* = 53). LOS was 1.54 ± 0.36 days after uncomplicated LAHR (*n* = 45) and 4.25 ± 0.71 days when bowel resection/anastomosis was required (*n* = 8). Time to enteral feeding followed a similar pattern: 14.13 ± 14.45 h overall (*n* = 53), 8.2 ± 1.7 h after uncomplicated LAHR (*n* = 45), and 47.5 ± 5.8 h in the resection/anastomosis subgroup (*n* = 8). This clear difference reflects the greater physiological impact and postoperative care requirements associated with bowel resection in complicated intussusception cases.

Regarding complications, post operative intraperitoneal infection occurred in 1 (1.89%, 95% CI: 0.33%–9.94%) patient which appeared as thick yellowish discharge from the intra-abdominal pelvic drain. There was no intraoperative or radiologic evidence of perforation. The patient improved with conservative management (temporary NPO, antibiotics, and drain care), and output resolved within 24 h.

Postoperative ileus occurred in 2 (3.77%, 95% CI: 1.04%–12.75%) patients, and wound infection occurred in 1 (1.89%, 95% CI: 0.33%–9.94%) patient. No cases of early recurrence within 48 h were observed in either group during the hospital stay or routine ultrasound evaluation prior to discharge.

## Discussion

Among patients underwent laparoscopic-assisted hydrostatic reduction (LAHR) after failed ultrasound-guided hydrostatic reduction (USGHR), the success rate was 84.91% (45/53), with 8/53 (15.09%) required mini-laparotomy with resection and anastomosis due to mass, perforation, or gangrene. No intraoperative iatrogenic perforations occurred. Postoperative complications were low (intraperitoneal infection 1.89%, ileus 3.77%, wound infection 1.89%), enteral feeding started at 8.2 ± 1.7 h, and the mean hospital stay was 1.54 ± 0.36 days. Under our standardized early-recurrence surveillance (≤ 48 h), no early recurrences were detected.

These findings support the feasibility and clinical utility of LAHR, but should be interpreted with caution given the single-arm design, modest sample size, and short in-hospital follow-up. In particular, recurrence surveillance was restricted to the immediate in-hospital period; late post-discharge recurrences were not systematically captured.

Laparoscopy offers real-time visualization of reduction, assessment of vascularity, and the ability to perform resection/anastomosis when required. Performing hydrostatic reduction under laparoscopic guidance adds a safety margin by minimizing bowel handling while allowing continuous inspection [11].

Our results align with reports evaluating laparoscopic-assisted or laparoscopic reduction after failed enema. Hamed et al. reported feasibility in 29% of attempted cases with a 75% success rate, comparable to our experience [13]. Chandrasekharam et al. documented short hospital stays, reinforcing the recovery benefits of minimally invasive approaches [14]. Apelt et al. found iatrogenic perforation to be rare (0.4%), consistent with our absence of intraoperative perforations [12]. Collectively, these studies support LAHR as a safe, effective alternative to open surgery following failed hydrostatic reduction [12–14].

Compared with laparoscopy alone, LAHR may confer advantages. The hydrostatic component provides gentle, pressure-driven reduction that limits traction and direct manipulation, potentially reducing serosal injury and perforation risk. Under laparoscopic vision, completeness and safety of reduction can be confirmed in real time. Apelt et al. reported a 0.4% perforation rate with laparoscopic-assisted techniques [12], whereas Wu et al. noted higher traction-related injury risk with purely laparoscopic reduction [9]. Chandrasekharam et al. also reported short stays and rapid feeding [14], outcomes comparable to our series.

Across methodologically comparable series in which hydrostatic reduction is performed under laparoscopic guidance after failed enema, outcomes consistently show high reduction success, very low iatrogenic perforation, and favorable recovery profiles, mirroring our experience [12–14]. Where laparoscopic-only series report higher traction-related injury risk, adding hydrostatic pressure under direct vision provides a plausible safety mechanism by minimizing manipulation [9]. Technique and timing (hydrostatic reduction under laparoscopy after failed USGHR) likely account for between-study differences and support LAHR as a safe, effective minimally invasive alternative to open surgery in this setting [12–14].

The combined technique also offers practical benefits by merging controlled hydrostatic pressure with precise visualization. Potential risks remain: excessive hydrostatic pressure may endanger ischemic bowel, and marked distension may limit visualization. Standardizing enema height/pressure, optimizing port placement, and close surgeon–radiologist coordination is therefore essential to maximize safety and reproducibility [9, 12].

Reporting the site of the intussusceptum anatomically within the colon (ileocecal/ascending, transverse, descending, sigmoid, rectosigmoid) facilitates comparison with other series that use anatomical descriptors [1, 13]. Length of stay in our cohort was comparable to Chandrasekharam et al. supporting consistent, rapid recovery with minimally invasive reduction [14].

These advantages remain preliminary. The study was neither randomized nor comparative and cannot establish superiority over other operative strategies. The absence of early recurrence is notable and suggests durable reduction under a standardized protocol, but longer follow-up is needed.

Time to enteral feeding (8.2 ± 1.7 h) compares favorably with prior reports. Chandra et al. observed time to feeds ≤ 24 h in most cases [8], and Hill et al. reported a median of 1 day to full feeds. Complication rates were low, and no intraoperative perforations occurred, supporting the added safety of combining hydrostatic pressure with laparoscopic visualization [15]. Apelt et al. similarly reported a 0.4% intraoperative perforation rate and low postoperative complications [12].

Routine short-course antibiotic prophylaxis may have contributed to the low infection rate, although this practice differs from selective, indication-based use recommended in many settings. While our institutional policy was driven by concerns of bacterial translocation and local infection-control protocols, we acknowledge that selective, indication-based antibiotic use could be equally safe and more consistent with global guidelines. Future protocols should evaluate whether limiting antibiotics to complicated cases maintains comparable safety [10, 12]. Likewise, routine oral antibiotics at discharge may be unnecessary in uncomplicated cases; although reflective of our institutional infection-control policy, may not be necessary in uncomplicated cases. Several guidelines and systematic reviews emphasize that selective, indication-based antibiotic use (limited to patients with perforation, peritonitis, or resection) can achieve comparable safety outcomes [10, 12]. Aligning with these standards in future protocols could minimize unnecessary antibiotic exposure while maintaining low postoperative infection rates.

### Limitations

This study is limited by its small sample size, short follow-up for most patients, and single-center design. A concurrent open-surgery control group was not included for ethical and practical reasons: because LAHR is minimally invasive with lower morbidity, randomizing patients with failed USGHR to open surgery was inappropriate where laparoscopy was available and standard. Outcomes were instead contextualized against historical and literature-based open cohorts, which typically show longer hospital stays, delayed enteral feeding, and higher complication rates; these indirect comparisons should be interpreted with caution. No subgroup analysis was performed to identify predictors of LAHR success or failure, as the LAHR cohort (*n* = 53) lacked sufficient power for meaningful stratification. Larger, multicenter studies with longer follow-up are needed to improve statistical power, generalizability, and evaluation of long-term complications.

## Conclusions

Taken together, these data suggest that LAHR provides a safe and effective alternative to laparoscopy alone after failed USGHR, particularly in cases with edematous or friable bowel. Compared with prior heterogeneous reports, our study contributes a protocolized, pressure-standardized LAHR pathway with milestone-based discharge, a prospectively defined early-recurrence window (48 h; none observed), and stratified recovery outcomes by resection status—offering reproducible methods and actionable targets for multicenter comparison and stewardship. While encouraging, these advantages should be considered preliminary; well-designed multicenter comparative studies (randomized where feasible or carefully matched where not) are needed to determine comparative superiority and refine standardized pressure protocols and antibiotic stewardship.

## Data Availability

Data is available upon reasonable request from corresponding author.

## References

[1] Hwang J, Yoon HM, Kim PH, Jung AY, Lee JS, Cho YA. Current diagnosis and image-guided reduction for intussusception in children. Clin Experimental Pediatr. 2022;66(1):12. 10.3345/cep.2021.01816 PMC9815940 35798026

[2] Vakaki M, Sfakiotaki R, Liasi S, Hountala A, Koutrouveli E, Vraka I, et al. Ultrasound-guided pneumatic reduction of intussusception in children: 15-year experience in a tertiary children’s hospital. Pediatr Radiol. 2023;53(12):2436–45. 37665367 10.1007/s00247-023-05730-6

[3] Attoun MA, Albalawi SMD, Ayoub A, Alnasser AK, Alkaram EH, Khubrani FA, et al. The management of intussusception: A systematic review. Cureus. 2023;15(11):e49481. 38152810 10.7759/cureus.49481 PMC10752083

[4] Alnamshan M, Almatroudi D, Almagushi DAL, Almadhi NA, Alenazi L. Idiopathic intussusception in infants and children: different outcomes in relation to interventions. Cureus. 2023;15(10):e48026. 38034175 10.7759/cureus.48026 PMC10688190

[5] Kiblawi R, Zoeller C, Zanini A, Kuebler JF, Dingemann C, Ure B, et al. Laparoscopic versus open pediatric surgery: three decades of comparative studies. Eur J Pediatr Surg. 2022;32(1):9–25. 34933374 10.1055/s-0041-1739418

[6] Younes A, Lee S, Lee JI, Seo JM, Jung SM. Factors associated with failure of pneumatic reduction in children with Ileocolic intussusception. Child (Basel). 2021;8(2):98–113. 10.3390/children8020136 PMC7918438 33673183

[7] Yang J, Wang G, Gao J, Zhong X, Gao K, Liu Q, et al. Liberal surgical laparoscopy reduction for acute intussusception: experience from a tertiary pediatric Institute. Sci Rep. 2024;14(1):457–96. 38172223 10.1038/s41598-023-50493-7 PMC10764731

[8] Chandra N, Dey SK, Narwar P. Management of intussusception in children: A comparative study of hydrostatic reduction with saline under ultrasound guidance versus laparoscopic assistance. Afr J Paediatr Surg. 2023;20(3):171–5. 37470551 10.4103/ajps.ajps_20_22 PMC10450121

[9] Wu P, Huang P, Fu Y, Lv Y, Feng S, Lou Y. Laparoscopic versus open reduction of intussusception in infants and children: A systematic review and meta-analysis. Eur J Pediatr Surg. 2022;32(6):469–76. 35688449 10.1055/s-0042-1749437

[10] Kelley-Quon LI, Arthur LG, Williams RF, Goldin AB, St Peter SD, Beres AL, et al. Management of intussusception in children: A systematic review. J Pediatr Surg. 2021;56(3):587–96. 33158508 10.1016/j.jpedsurg.2020.09.055 PMC7920908

[11] Fahiem-Ul-Hassan M, Mufti GN, Bhat NA, Baba AA, Buchh M, Wani SA, et al. Management of intussusception in the era of ultrasound-guided hydrostatic reduction: a 3-year experience from a tertiary care center. J Indian Assoc Pediatr Surg. 2020;25(2):71–5. 32139983 10.4103/jiaps.JIAPS_208_18 PMC7020677

[12] Apelt N, Featherstone N, Giuliani S. Laparoscopic treatment of intussusception in children: a systematic review. J Pediatr Surg. 2013;48(8):1789–93. 23932624 10.1016/j.jpedsurg.2013.05.024

[13] HAMED MS, MAGED EK. Prospective evaluation of laparoscopy in management of infantile intussusception. Med J Cairo Univ. 2019;87(March):653–9.

[14] Chandrasekharam VV, Gazula S, Gorthi RP. Laparoscopy-assisted hydrostatic in situ reduction of intussusception: A reasonable alternative? J Indian Assoc Pediatr Surg. 2011;16(1):8–10. 21430840 10.4103/0971-9261.74513 PMC3047778

[15] Hill SJ, Koontz CS, Langness SM, Wulkan ML. Laparoscopic versus open reduction of intussusception in children: experience over a decade. J Laparoendosc Adv Surg Tech A. 2013;23(2):166–9. 23327343 10.1089/lap.2012.0174

